# Harnessing ontology and machine learning for RSO classification

**DOI:** 10.1186/s40064-016-3258-2

**Published:** 2016-09-26

**Authors:** Bin Liu, Li Yao, Dapeng Han

**Affiliations:** 1 Science and Technology on Information Science and Engineering Laboratory, National University of Defense Technology, Changsha, 410073 People’s Republic of China; 2 College of Aerospace Science and Engineering, National University of Defense Technology, Changsha, 410073 People’s Republic of China

## Abstract

Classification is an important part of resident space objects (RSOs) identification, which is a main focus of space situational awareness. Owing to the absence of some features caused by the limited and uncertain observations, RSO classification remains a difficult task. In this paper, an ontology for RSO classification named OntoStar is built upon domain knowledge and machine learning rules. Then data describing RSO are represented by OntoStar. A demo shows how an RSO is classified based on OntoStar. It is also shown in the demo that traceable and comprehensible reasons for the classification can be given, hence the classification can be checked and validated. Experiments on WEKA show that ontology-based classification gains a relatively high accuracy and precision for classifying RSOs. When classifying RSOs with imperfect data, ontology-based classification keeps its performances, showing evident advantages over classical machine learning classifiers who either have increases of 5 % at least in FP rate or have decreases of 5 % at least in indexes such as accuracy, precision and recall.

## Background

The resident space object (RSO) identification is a main focus of space situational awareness (SSA) (Linares et al. 2014; Nebelecky et al. 2014; Henderson 2014), and also an important task for space agencies, where RSO classification plays an important role (Ruttenberg et al. 2015). Current RSO classification aims at classifying RSOs such as satellites, space stations, debris and rocket bodies in earth orbits, based on their features extracted from observations. Automatic RSO classification helps to continuously update the knowledge of the RSOs’ status such as types, missions, and even detailed characterizations, which is required by SSA (Cox et al. 2016). Accurate, robust and comprehensible classification of RSO in open environments imporves efficiency of the analysts of SSA to make decisions.

Modeling RSO for accurate classification with the aid of machine learning has aroused interests of more and more researchers (Wang et al. 2012; Poole and Murray-Krezan 2015; Howard et al. 2015), as the collected RSO data accumulate. Machine learning has been widely used for its advantage in discovering knowledge from high-dimension data, which are difficult for human to analyze. With the specific knowledge discovered by machine learning from the data closely related to scenario, classification accuracy can be greatly enhanced. However, the collected RSO data are often noisy and incomplete, making it difficult for machine learning to acquire some “common sense” of experts just by analyzing the noisy and incomplete data (Sakurada et al. 2015). Moreover, overfitting problems are common for machine learning (Sakurada et al. 2015). These limitations make machine learning classifiers unable to classify RSOs correctly, especially when some features are missing because of the limited and uncertain observations (e.g., only poor telescope observations). Besides, due to the limited observations, it remains a difficult task to identify the type and function of an RSO (Ruttenberg et al. 2015). In addition, the comprehensibility of RSO classification also shall be concerned, for the understanding of SSA technologies helps analysts to make decisions (Ianni et al. 2012; Haith and Bowman 2014; Poole and Murray-Krezan 2015).

Researchers have embedded background knowledge into machine learning classifiers, aiming at improving robustness (Al Momani et al. 2006; Lauer and Bloch 2008; Sinha and Zhao 2008; Li et al. 2009; Orchel 2011), or improving classification accuracy (Silva and Ribeiro 2009). If background knowledge is used in RSO classification, it can also improve the robustness of classification in the existence of missing features. For example, hierarchical RSO classification (Ruttenberg et al. 2015) is able to classify an RSO with low specificity but more correctness when some features of the RSO are missing. It is better to classify a remote sensing satellite as an earth observation satellite than to classify it as a navigation satellite, for earth observation satellite is a type of remote sensing satellite and navigation satellite is not. It is also convenient to utilize different feature sets for classification on different hierarchies. Therefore, RSO hierarchy can play significant roles in RSO classification with imperfect data. Another example is deducing missing features from given features according to background knowledge—some missing features of an RSO can be estimated or calculated from known features according to experience or models, then the RSO is classified with high specificity, using the known features and the deduced features.

Recently, ontology-based classification (OBC) is applied to more and more classifications such as documents classification and emotion recognition in web (Song et al. 2006; Zhang et al. 2013), classifications of adverse drug reactions and epilepsy types in clinical (Zhichkin et al. 2012; Kassahun et al. 2014), harbor surveillance (Gómez-Romero et al. 2015), building classification in remote sensing (Belgiu et al. 2014), complex object recognition in cognitive vision (Maillot and Thonnat 2008), chemicals and molecules classifications (Magka 2012; Hastings et al. 2012), etc. Summarizing the works listed above, one can see the common reasons for choosing OBC. Firstly, ontology is a suitable way to represent background knowledge such as contextual knowledge (Hong and Nugent 2013; Gómez-Romero et al. 2015). Secondly, OBC is an optional way to handle classification problems for the advantage of integrating heterogeneous data (Wang et al. 2009; Gómez-Romero et al. 2015). Thirdly, owing to the integration of rules, an OBC system can be formed through integrating background knowledge and machine learning rules conveniently using ontology, bringing advantages including enhanced transparency (Lüscher et al. 2009) and augmented comprehension, easy representation and enhanced flexibility (Lüscher et al. 2009) such as convenient modification (Paul Suganthan et al. 2015) for robust and comprehensible RSO classification.

In this paper the authors propose a method to construct an OBC system for RSO through integrating unordered machine learning rules and background knowledge. Rules are unordered, meaning that the prediction made by each rule is independent of the applicability of other rules (Han et al. 2011). By background knowledge it means RSO hierarchies and domain knowledge related to the RSO classification. There are various types of domain knowledge for RSO classification. Knowledge describes RSO themselves, e.g. physical characteristics of the RSO, is called *internal knowledge*. Knowledge that is about external information which completes, influences or constrains the operations of the RSO, or knowledge about the expected behavior of RSOs, is called *contextual knowledge*. For example, knowledge about physical characteristics of the environment such as atmospheric drag, knowledge about solar radiation pressure and gravity of the RSOs in orbit, expectation of a specific satellite’s attitude, belong to *contextual knowledge*. There is also knowledge used for deducing features based on given features. In the following, the term *deduction knowledge* will be used to identify the knowledge by which more information about the RSO can be inferred. For example, given the RSO’s length and radius of its bottom circle, the volume’s formula of an RSO with the form of cylinder is one kind of *deduction knowledge*.

The OBC system’s goal is achieved by the following steps. Firstly, an ontology for RSO classification named OntoStar is built upon the background knowledge and unordered machine learning rules of RSO. Secondly, the system applies deductive and rule-based reasoning to extend tracking data, and to classify RSO according to its features collected by authorized organizations such as North American Air Defense Command (NORAD) and Union of Concerned Scientists (UCS). The source of the knowledge for classification in OntoStar is traceable, and the inference of classifying an RSO is recorded, making the classification explainable and comprehensible.

To the best of our knowledge, this is the first attempt to classify RSOs using OBC, especially OBC that integrates unordered machine learning rules and background knowledge. And it is also the first exploration of extra awards of OBC, such as the traceable and comprehensible classification. Similar approaches in the literature have focused on alternative probabilistic models such as probabilistic programming (Ruttenberg et al. 2015), or the ontologies’ application to classifications (Song et al. 2006; Zhichkin et al. 2012; Magka 2012; Belgiu et al. 2014; Zhang et al. 2013; Kassahun et al. 2014; Gómez-Romero et al. 2015; Hastings et al. 2012). Ontology facilitates the creation of a computable model representing complex situational context (problem entities, entities relationships, etc.) and human knowledge from experience and theory, since they can be formally encoded in a logic-based expressive language. The examples show that OBC improves performances with respect to machine learning classifiers, and keeps its performance while machine learning classifiers’ performances reduce drastically when some features are missing. Furthermore, OBC gives traceable and comprehensible reasons for RSO classification, so every step of the classification can be validated by experts.

The rest of this paper is organized as follows. “Related works” section discusses related work in OBC, together with background knowledge for RSO classification and unordered machine learning rules. “OntoStar development” section studies the ontology development for RSO classification. “OntoStar development” section presents experiment results of applying OBC in RSO classifications, giving comparison to results of machine learning classifications for RSO on WEKA^1^ platform, and illustrating additional advantages of OBC. The paper concludes with discussions on the contributions of the work and directions for future research in “Conclusions” section.

## Related works

### Ontology-based classification

During the last decade, OBC has been quite extensively applied in classification. It is considered as a complement for existing classification algorithms, owning at least four extra awards (Belgiu et al. 2014), which are: (1) consistency checking with tools support; (2) a declarative model which can be scrutinized by peers and extended to other application scenarios; (3) inferred implicit knowledge by reasoning; (4) representations which can be understood by both machine and human, and as a result, assessment validation of the deduction can be made by users. Besides these advantages, OBC is convenient to integrate rules, and rule-based classifiers have advantages such as augmented comprehension, easy representation and convenient modification (Paul Suganthan et al. 2015).

**Fig. 1:**
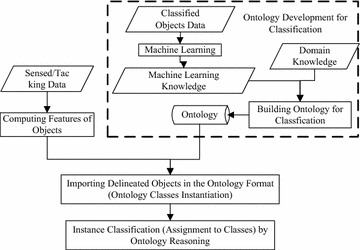
General architecture and computational process of OBC

In most applications of OBC, classifications are mainly realized through instance classification, which is based on two processes. First, the ontology for OBC is built. Then, when a new object is created or its property values are modified in the ontology, a reasoning process is applied to the ontology to find matches to the descriptions of the object, and determines the class(s) which the new object belongs to. The architecture and computational process of OBC are generally similar to the description shown in Fig. 1.

In the first step, namely *development of ontology for classification*, formally represented knowledge can and shall be reused as the domain knowledge. Knowledge sharing and reuse are addressed in developing ontologies (Gruber 1993). Using shared specifications makes OBC understood by domain experts and the reuse of domain knowledge enhances efficiency of developing the ontology for classification. Other criteria to guide the development of ontologies are also proposed (Gruber 1995; Haghighi et al. 2013; Suárez-Figueroa et al. 2015), which standardize the development of ontology. Characteristics of concepts can also be learned by machine learning and then used as definitions of the concepts. Maillot and Thonnat (2008), Belgiu et al. (2014) integrate machine learning knowledge into ontologies through this way. By representing machine learning rules through Semantic Web Rule Language (SWRL) (Horrocks et al. 2004), the machine learning knowledge can be integrated into ontologies in another way. Zhang et al. (2013) chooses this way to code machine learning knowledge into ontology. The latter way for integrating machine learning knowledge is convenient to record source of knowledge, being able to express more complex relations between objects.

In the last step, namely *instance classification*, reasoning can be performed by various reasoners, such as general reasoners used in Belgiu et al. (2014), Gómez-Romero et al. (2015), or the dedicated reasoner in Magka (2012).

Ontology development for classifications are the main concerns in former studies on OBC. However, there are more things that OBC can do. Sources of the knowledge for ontology development can be easily recorded in OBC. Transparent classification processes can be supported by existing tools, as can be seen in ontology explanation (Kalyanpur et al. 2007) implemented in pellet.^2^ The traceable knowledge and transparent classification are able to make the classifying results more comprehensible and convincing, with every step of the classification can be validated. In addition, experiments show that classification accuracy can be further enhanced by combining induction and deduction, as one can obtain consistent deductive knowledge through consistency checking for inductive results (Lécué and Pan 2015).

### Background knowledge for RSO classification

Background knowledge can be defined as information about the topics being dealt with, or information regarding the domain under study. It provides explicit information about domain. In terms of classifications, background knowledge can be concept hierarchy (Santoso et al. 2011), readily available information improving classification accuracy, knowledge related to classifications, or even test examples used to aid in classifications (Zelikovitz and Hirsh 2001). In the OBC system for RSO classification, there are two types of background knowledge—hierarchies and domain knowledge related to the classification. RSO hierarchies, data and domain knowledge related to classification will be the main focuses in the following part.

#### RSO classification, hierarchies and data

There are two main types of existing models for RSO classification. One is the type of models that are built on features extracted from single-source observations. The other is the type of models that are based on RSO hierarchies. Under effective observations, specific types of RSO can be classified with features extracted from single-source data by machine learning classifiers. For example, six features are extracted from photometric data of RSO in Howard et al. (2015), and then several machine learning classifiers are built on the data of the 6 features to classify RSOs; statistical values, size and attitude are extracted from narrow band radar in Wang et al. (2012), and a KNN fuzzy classifier is learned from the extracted features. However, it is limited to classify multiple types of RSOs based on single-source observations, especially when features for classification are missing because of uncertain observations. Therefore, multi-source data have to be collected to get a larger candidate feature set in some cases for robust classification. Then it is possible for classifiers to utilize different feature sets for classifications on different RSO hierarchies, with RSO being classified with low specificity even if missing values exist.

Concept hierarchies express the structure of concepts from specific to general. They allow expressing the discovered knowledge in a higher abstraction level, more concise format (Di Beneditto and de Barros 2004), hence facilitate effective reasoning for OBC. Concept hierarchies are mostly built by experts. Expert-built hierarchies are always built based on a series of key features or attributes. The most well-known and widely accepted expert-built concept hierarchy is the classical Linnaeus biological taxonomy (Godfray 2007). There are also concept hierarchies built by machines. For example, concept hierarchies can be built from databases when they may be implicit within the database schemas and their instances (Trinkunas and Vasilecas 2007). As a kind of background knowledge, concept hierarchies provide information which can be used to guide hierarchical classification (Ruttenberg et al. 2015), or used to classify objects when expressing the discovered knowledge for classification in concept form (Belgiu et al. 2014).

In the RSO classification domain, there are already some expert-built RSO hierarchies. Fruh et al. (2013) characterizes RSO with six key discrete features related to RSO such as orbit parameters, materials and shape, and constructs an artificial RSO hierarchy based on these features. Similar to the proposal of Fruh et al. (2013), using four features of RSO (intrinsic information) such as size, attitude, shape and spin control (Linares et al. 2014), builds a four-layer RSO hierarchy. Wilkins et al. (2013) builds an RSO hierarchy using several external information of RSO such as orbit class and manufacturer of RSO. Ruttenberg et al. (2015) classifies RSO hierarchically by reasoning on an RSO hierarchy using probability programming, and deduces the features needed for further classifications to drive RSO observation; 50 satellites have been chosen from UCS_Satellite^3^ to validate feasibility of the method, however, the competence of the model and completeness of knowledge in the model need to be further validated. Taxonomies of RSO are also contained in domain ontologies, such as in Rovetto (2016), Rovetto and Kelso (2016), Cox et al. (2016).

These expert-built RSO hierarchies provide us very good materials for building OntoStar. However, descriptions of concepts in these hierarchies are single and relatively simple, making classification models based on these hierarchies unable to function properly when some key features are missing. Descriptions of concepts in these hierarchies can be further extended with the learning knowledge from RSO data, in order to classify RSO under limited and uncertain observations. There are several dependable data sources of RSO, such as the dataset Norad_Catalog of website Satellite_Debris,^4^ the dataset UCS_Satellite of UCS, the RSO data published by calsky.com,^5^ TLE data published by Spacetrack.org.^6^ Norad_Catalog dataset describes *radar cross section (rcs)*, *orbit*, and *area*-*to*-*mass ratio (amr)* etc. of RSO, and labels RSO as *Satellite*, *Debris* and *Rocket Body* (abbr. *Rocket* in the following). UCS_Satellite dataset describes active satellites with *mass*, *power*, *purpose* and so on. The calsky website publishes *radar cross section*, *shape*, *magnitude* and *size* of RSO. TLE data published by Spacetrack.org describes *orbit* of RSO. With the data containing implicit concept hierarchies with labels, reverse engineering methods can be utilized to obtain explicit RSO hierarchy, and machine learning can be used for descriptions of the concepts.

#### Domain knowledge related to RSO classification

As pointed out above, there are three types of domain knowledge useful to the RSO classification: *internal knowledge*, *contextual knowledge* and knowledge used for deducing features based on given features (called *deduction knowledge*).

Models describing characteristics of RSO have been studied quite extensively. There are many models that specify the characteristics of different types of RSO from different perspectives, such as models of space object (Han et al. 2014; Henderson 2014; Howard et al. 2015; Poole and Murray-Krezan 2015) and debris (Rovetto 2016), material density distribution model of space debris (Opiela 2009). The distinct characteristics of a specific type of RSO can be used as *internal knowledge* of RSO to distinguish the type of RSO from others, for example, photometric signatures can be used to classify rocket body and communication satellite (Howard et al. 2015). There are also domain ontologies of RSO, which studies specification of RSO and relations between RSO and their relative entities, such as Rovetto (2016), Rovetto and Kelso (2016), Cox et al. (2016).

There is much *contextual knowledge* of RSO scattered in models, such as relations between satellite and related concepts described in the Space Surveillance Ontology (Pulvermacher et al. 2000), expected attitudes or orbit control of specific types of satellites (Wilkins et al. 2013). Contextual knowledge can also be used for RSO classification, for example, satellites of particular contractors are expected to be for particular missions (Wilkins et al. 2013).

*Deduction knowledge* is deployed to deduce new feature/attribute data which may be required in the RSO classification, from some known features/attributes. When some attributes of RSO are unknown under limited or uncertain observations, the estimated values of the attributes deduced by *deduction knowledge* are always more accurate than estimated values achieved merely via statistics, because *deduction knowledge* has to be validated and tested through practice. *Deduction knowledge* related to RSO classification is mainly from experience and literature. For example, the formula of computing the volume given a cylindrical RSO’s length and radius of its bottom circle comes from experience, and the formula for estimating *area*-*to*-*mass ratio* of an RSO given its orbital parameters comes from literature (Espitia and Schulz 2013). Moreover, one can also mine implicit *deduction knowledge* from data such as mining relations between objects (Gal et al. 2013) and deriving data of satellites on a database (Mansinghka et al. 2015).

### Unordered machine learning rules for OBC

Ontologies for classifications, when constructed manually, are always far from complete, hence restrict the competences of OBC systems. Competences of OBC systems for RSO can be enhanced through the integration of rules learned from the collected RSO feature data. As stated in “Ontology-based classification” section, SWRL are more expressive and can record sources of knowledge, machine learning knowledge are integrated into the OBC system for RSO, in the way of adding SWRL rules transformed from machine learning rules to ontology.

Two types of rules have traditionally been considered in rule learning (Han et al. 2011): decision lists (or ordered rule sets), and rule sets (unordered rules). The ordered rule sets are more compact and generally can generate higher ROC curve. However, the unordered rules are more suitable for classification scenario for three reasons. Firstly, it is rather easy to modify a model based on unordered rules, for adding or removing a rule from the model will not influence other parts of the model, making it easy to add background knowledge as unordered rules to the model. Secondly, SWRL rules for classifications can be added to the ontology directly with no influence on other parts of the model, which consequently improves the competence of the OBC system. Thirdly, SWRL rules supported by existing ontology reasoners are unordered.

Unordered rules can be obtained directly from rule generators such as MCNN (Alpaydin 1997), TFPC (Coenen et al. 2005), and can also be obtained by extracting rules from other classification models such as decision trees learned by C4.5 (Han et al. 2011). These rules can be easily transformed to SWRL rules. However, redundancy and inconsistency between them have to be handled properly, before they are integrated to ontologies. There are ways to remove redundant rules, such as semantic pruning to get representative association rules (Ferraz and Garcia 2013), or association rule extraction (Xu et al. 2015). Ways can be tried to handle inconsistency. For example, a classification can be decided by voting, when multiple classes are deduced (Han et al. 2011). Also, sometimes it is nice to accept only classification results which are consistent with domain ontologies (Lécué and Pan 2015).

## OntoStar development

OntoStar is the basis of an OBC system for RSO. This section firstly presents a scheme to code background knowledge and unordered machine learning rules into OntoStar with the Ontology Web Language (OWL) (Group 2007) and SWRL. Then it will be described how rules can be learned for OntoStar.

### Integrating ontology and unordered rules for classification

As introduced in “Background” section, OntoStar consists of background knowledge and machine learning rules. The background knowledge such as concept hierarchy, *internal knowledge* and *contextual knowledge* of concepts in OntoStar, is coded with OWL.

According to the ontology definition in Gruber (1993), there maybe multiple ontologies for the same concept depending on the purposes for modelling ontologies. The purpose of the authors is to model a task ontology for the classifications of RSO named OntoStar. There are domain ontologies about RSO (Rovetto 2016; Rovetto and Kelso 2016; Cox et al. 2016) and databases of RSO such as Norad_Catalog and UCS_Satellite, which can be reused and extended by OntoStar. Relevant concepts are extracted from the domain ontologies. The hierarchy of OntoStar are derived from the domain ontologies and databases. By way of example, the direct subclasses of RSO (*SpaceObject*)—*Rocket* (*Rocket Body*), *Debris* and *Satellite*, are referred to Norad_Catalog, and the taxonomy of *Satellite* is derived from UCS_Satellite based on the purposes of satellites. In Rovetto and Kelso (2016), specifications of RSO of different disciplines are clearly stated. There are also other concepts which are related to RSO classification, such as *orbit*, *payload* and concepts of features. Relations of RSO are specified in Cox et al. (2016).

The instances of RSO can be described by object properties and instances of the related concepts. For example, given an instance of *SpaceObject*: International Space Station *iss*, which has a payload *CATS*. Then *iss* can be described by the instance of *Payload*—*CATS*, as the form “hasPayload(iss, CATS) ∧ Payload(CATS)”.

**Fig. 2:**
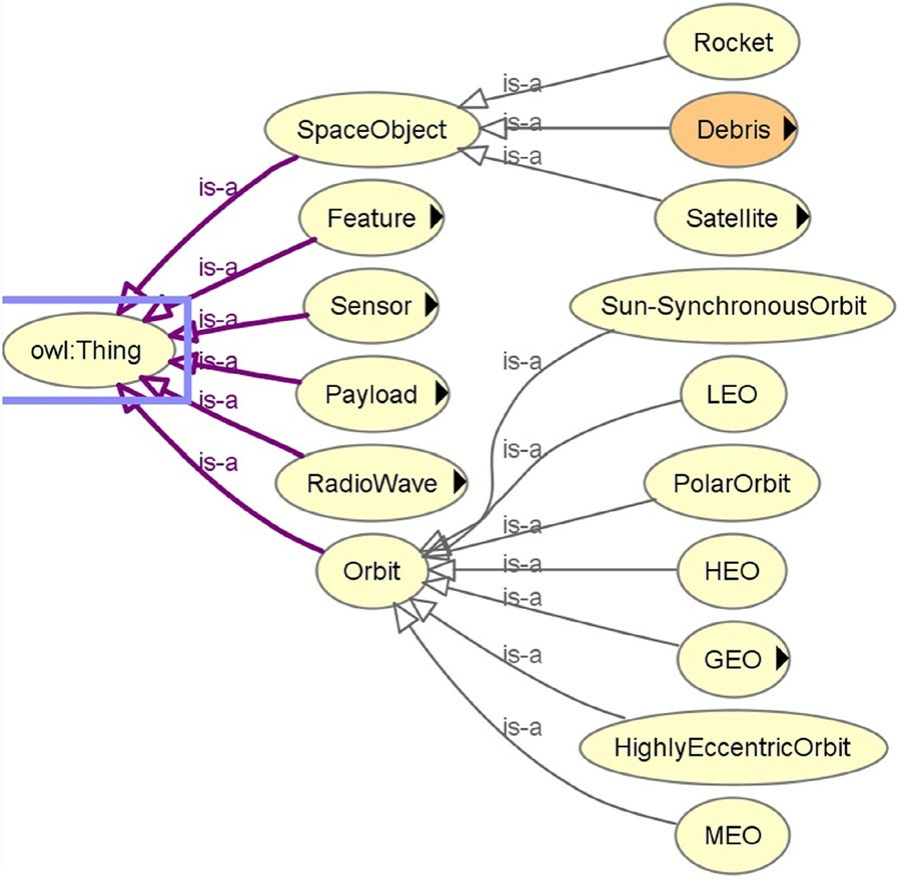
Part of the hierarchy in OntoStar

The top two concepts of RSO (named *SpaceObject* in Fig. 2) and *Orbit*, and the other top concepts related to RSO classification of OntoStar are shown in Fig. 2.

The *internal knowledge*, together with *contextual knowledge* of concepts in OntoStar, is used to describe concepts in OntoStar, and may also be used to classify instances of RSO. For example, the concept *Debris* are divided according the feature *size*. So *size* is the intrinsic of *Debris*, and the knowledge describing a subclass of *Debris* is *internal knowledge* of the subclass. Table 1 shows examples of the above knowledge coded with OWL in OntoStar.

**Table 1:** examples of knowledge coded with OWL in OntoStar

Knowledge type	Example
Concept hierarchy	LargeDebris SubClassOf Debris
	Debris SubClassOf SpaceObject
	Rocket SubClassOf SpaceObject
	Satellite SubClassOf SpaceObject
	Disjoint Class (Debris Rocket Satellite)
*Internal knowledge*	LargeDebris≡Debris and (size only xsd:float[>0.1f])
*Contextual knowledge* of concepts	SpaceObject SubClassOf (inOrbit exactly 1 Orbit)
	HEO≡Orbit and altitude only xsd:float[>=36000.0f]
	Orbit SubClassOf (inclination exactly 1 xsd:float[>=0f,<180])

A 3-layer concept hierarchy is shown in Table 1, listed as *RSO*-*Debris*-*LargeDebris* from general to specific. The subclasses of concept *SpaceObject* (Debris |Satellite |Rocket) are disjoint with each other. The concept *LargeDebris* is defined by its characteristic of *size*, for example, *Debris* with a *size* larger than 0.1 (m) is *LargeDebris*. The first constraint of the RSO’s operations is that every RSO is in an *Orbit*. The concept *Orbit* can be classified by its *altitude*, and is a *HEO* when its *altitude* is not less than 36,000 (km). *Orbit* has the description of *inclination* whose value is in the range of [0,180).

The background knowledge in OntoStar, such as *deduction knowledge* and other *contextual knowledge*, is expressed in unordered rules called *background rules*. Machine learning rules in OntoStar are also unordered. Every *background rule* or machine learning rule is coded with SWRL, containing annotations of additional information such as the rule’s source and evaluations (if exist). Machine learning knowledge is coded with SWRL, other than defining concepts with the learning features like the way in Maillot and Thonnat (2008), Belgiu et al. (2014), for the reasons mentioned in “Ontology-based classification” section, and also for the reason that rules extracted from decision trees are more comprehensible than decision trees, particularly when the decision trees are very large (Han et al. 2011).

Only rules extracted from C4.5 are chosen to be integrated into OntoStar for the following reasons. Firstly, an implementation of C4.5 named J48 is provided by WEKA, and rules extracted from C4.5 are unordered rules. Secondly, redundancy and inconsistency may occur between multi-source unordered rules, and there are no redundancy and inconsistency between rules extracted only from C4.5. To emphasize the main idea of this paper, redundancy and inconsistency are not addressed by this paper. Table 2 gives examples of SWRL rules expressed in Manchester Syntax (Horridge and Patel-Schneider 2008) in developing OntoStar.

**Table 2:** Examples of SWRL rules in the developing OntoStar

*Background rules*	Feature computational rules	so:SpaceObject(?S), so:shape(?S,?Shape), Cynlinder(?Shape), so:height(?S,?H), so:bottom_area(?S,?A), swrlb:multiply(?V,?A,?H) → so:volume(?S,?V) annotations: Source = Mathematics
		so:rcs(?X,?RCS), so:power(?P,?D,0.5f), so:SpaceObject(?X), swrlb:divide(?D,?RCS,0.79f), swrlb:subtract(?S,?P,2.57E-13f) → so:size(?X, ?S) annotations: Source = Experience
	Semantic classification rule	so:SpaceObject(?S), so:power(?S,?pv), swrlb:greaterThan(?pv,1.0f) → so:Satellite(?S) annotations: Source = Domain
Unordered machine learning rules	Rule from C4.5	so:Satellite(?S), so:MEO(?O), so:inOrbit(?S,?O), so:launchSite(?S,’Cape_Canaveral’) → so:Navigation_Satellite(?S) annotations: Source = mining from UCS_Satellite using J48 Confidence = 0.9873 Coverage = 26
		so:SpaceObject(?S), so:size(?S,?Sz), swrlb:lessThanOrEqual(?Sz,0.39), so:amr(?S,?A), swrlb: lessThanOrEqual(?A,0.01), so:inOrbit(?S,?O), so:eccentricity(?O,?E), swrlb:greaterThan(?E,0.001426), so:apogee(?O,?AP), swrlb:greaterThan(?AP,784), period(?O,?P), swrlb:greaterThan(?P,108) → so:Debris(?S) annotations: Source = mining from Norad_Catalog using J48 Confidence = 1 Coverage = 37

*Background rules* and unordered machine learning rules are shown in Table 2. There are three *background rules*.

The first rule is knowledge about computing volume of RSO with cylindric form. The rule can be read as following:

*the variable ?S is a space object of cylindric form, its height is ?H and its bottom area is ?A, it can be concluded that the volume of ?S equals to ?H multiplying ?A*.

The second rule is knowledge from experience that describes how to derive *size* using *radar cross section (rcs)*.

The third rule is *contextual knowledge* stating that an RSO with power greater than 1 (watts) is expected to be a satellite (not vice versa, i.e., not every satellite is with power greater than 1, thus, *power* is not the intrinsic of *Satellite* and this rule is only a background rule for classification rather than *internal knowledge*).

There are two unordered machine learning rules, which are extracted from two C4.5 decision trees respectively. The first rule is extracted from a C4.5 decision tree which is learned from the dataset UCS_Satellite. It has covered 26 samples. The second rule is extracted from a C4.5 decision tree derived from Norad_Catalog. It has covered 37 samples. The two rules are used for classifications of satellite and RSO respectively.

### Learning classification rules for OntoStar

As pointed out in “Unordered machine learning rules for OBC” section, it is necessary to obtain rules by learning, in order to improve performances of OBC system, with the additional advantage to enrich the descriptions of concepts in ontology for robust classification when some features are missing. Describing RSO with different feature sets makes the classification more flexible, and not dependent on only one feature set. Classification rules learned from features RSO data are just about descriptions of RSO with feature sets. When learned classification rules are integrated into OntoStar, the completeness of OntoStar increases.

In the presence of limited or uncertain observations, there are usually some features of RSO missing. Under this condition, it is better to classify an RSO with low specificity than not to classify it, and it is better not to classify an RSO than to classify it incorrectly. In order to get better results, RSOs classified with low specificity and unclassified RSOs are left by the OBC system to experts for further treatment. Therefore, when learning rules for OntoStar, every learning process corresponds to one hierarchy of OntoStar. That is to say, classification rules of RSO are learned hierarchically. This kind of learning is called *hierarchy guided learning*. For example, it first obtains the machine learning rule about the concept *SpaceObject* of the form (SWRL rules in Manchester Syntax)

$$SpaceObject(?S), \ldots \rightarrow Satellite(?S),$$

then it obtains the machine learning rule about the concept *Satellite* of the form

$$Satellite(?S), \ldots \rightarrow Communication\_Satellite(?S).$$

Besides, guided by the hierarchy, every learning process concentrates on a smaller range, and gains smaller decision trees. When deciding the more specific type of an RSO, only rules expressed in the form *SpaceObject*(?*S*), … → *X*(?*S*) (X∈{C |C is a subclass of SpaceObject}) are explored. So the searching space is expected to be smaller.

Once the classification rules are learned, they should be transformed to SWRL representation, so that they can be integrated into OntoStar, combining with background knowledge expressed by OWL and SWRL. Classification rules learning for OntoStar is summarized in Algorithm 1.
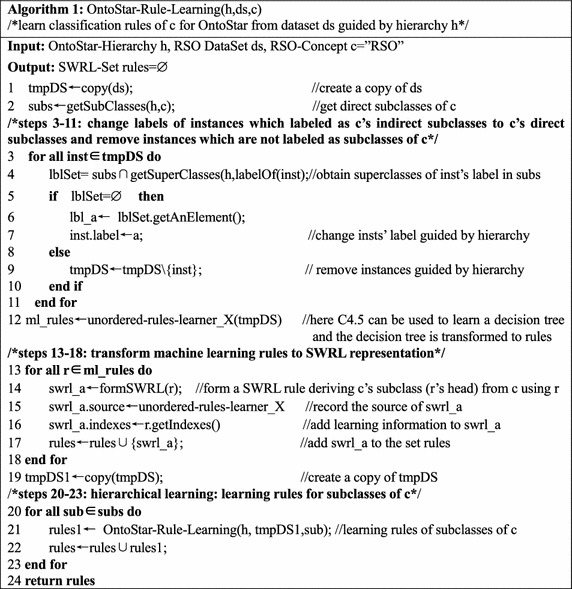


Algorithm 1 learns classification rules of the class ***c*** and its subclasses from dataset ***ds*** with the guidance of hierarchy ***h***. In steps 3–11, only instances of ***c*** are saved to ***tmpDS*** for learning. If there are no steps 3–11 and steps 20–23 in Algorithm 1, information of the ontology is not used in learning rules and rules are not learned hierarchically. This kind of rule learning for *classification ontology* is called *simple learning*. Continuing the example above Algorithm 1, rules of the following form will be obtained by simple learning

$$\begin{aligned} &SpaceObject(?S), \ldots \rightarrow Satellite(?S) \hfill \\ &SpaceObject\left( {?S} \right), \ldots \rightarrow Communication\_Satellite(?S). \hfill \\ \end{aligned}$$

*Simple learning* means domain knowledge and machine learning knowledge are simply combined for classification. *Simple learning* is useful, for machine learning can still be deployed when the hierarchy isn’t available or exact.

In step 12, rules of deriving direct subclasses of ***c*** from ***c*** are learned. In steps 13–18, machine learning rules are transformed to SWRL rules. It should be pointed out particularly that, the atom ***c(?X)*** should be added to the body of the transformed SWRL rules, because only instances of ***c*** are used when Algorithm 1 is employed. In steps 20–23, classification rules of the class ***c*** and its subclasses are learned by employing Algorithm 1 recursively.

## Experiments and result analysis

In this section, it will firstly be shown how multi-source data of RSO are represented by OntoStar. Then classification of RSO, transparent classification process and traceable knowledge are shown on Protégé. Afterwards, the OBC system is integrated into WEKA to test its performances, where the classification result is compared to that of classical machine learning classifiers. To be clarified, the OBC system specially designed for RSO is named R-OBC in the following.

### Representation of RSO by OntoStar

NORAD_Catalog and UCS_Satellite are collected for the experiments. NORAD_Catalog describes RSOs with the following attributes/features: *cospar_id*, *nord*_*id*, *period*, *perigee*, *apogee*, *eccentricity*, *rcs*, *amr* and labels. There are 8071 samples in this dataset with 3 labels: *Debris*, *Rocket Body* and *Satellite*. UCS_Satellite describes active satellites with the following attributes/features: *cospar_id*, *nord*_*id*, *period*, *perigee*, *apogee*, *eccentricity*, *orbit type*, *orbit class*, *longitude*, *power*, *dry mass*, *launch mass*, *launch vehicle*, *launch site*, *owner*, *contractor*, *users* and *purposes*. The attribute *purposes* is used as the dataset’s labelling attribute. The dataset contains 1346 samples with 19 labels. The dataset is incomplete, for it contains 1267 samples with at least one attribute of missing value. Data in NORAD_Catalog are used to analyze orbital distributions of different types of RSOs in Savioli (2015), and also used to derive new features’ data of satellites in Mansinghka et al. (2015). Data in UCS_Satellite are used by Ruttenberg et al. (2015) to describe some types of satellite.

The above two datasets contain 318 identical RSOs and have 9099 RSOs in total. To make all the RSOs able to be classified by classical classifiers, the two datasets are merged into one, through left join on the attribute *cospar_id*. The 318 identical RSOs are labelled the same as their labels in UCS_Satellite. Finally the dataset RSODS is obtained.

RSODS is an incomplete dataset, for it contains only 79 records without missing values. The dataset composes of records of *Debris* (61.6 %), *Rocket* (7.6 %), unknown-type *Satellite* (15.9 %) and *Satellite* of specific purposes (14.8 %) such as *Communication Satellite*, *Global Position Satellite*, *Earth Observation Satellite* and etc., adding up to 21 types of RSOs. All the 79 records without missing values are satellites of specific purposes.

**Fig. 3:**
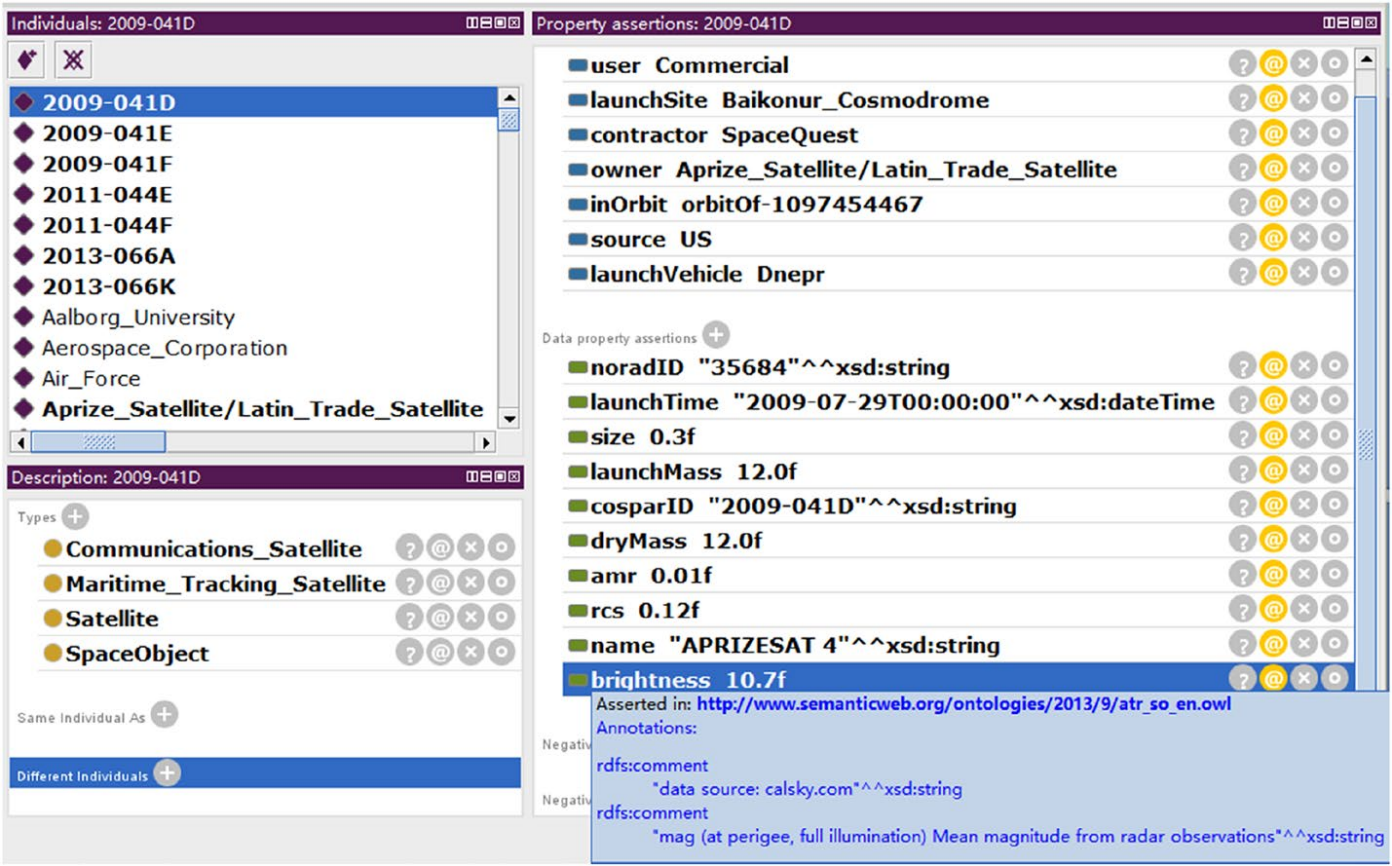
Description of *2009*-*041D* in OntoStar

Taking the RSO whose cospar_id is “2009-041D” as example, it will be shown how to describe RSO and record sources of the data by OntoStar. Firstly, an instance of *SpaceObject* is created in OntoStar named “2009-041D” (identified as *2009*-*041D* in the following), data in NORAD_Catalog and UCS_Satellite about *2009*-*041D* are used to describe the instance *2009*-*041D* in OntoStar correspondingly. Then brightness datum about *2009*-*041D* collected from calsy.com is used to fill the data property *brightness* of the instance *2009*-*041D*. The description of the instance *2009*-*041D* using the data from the three sources above is shown in Fig. 3.

In Fig. 3, the description of *2009*-*041D* is shown in Protégé,^7^ which is an integrated environment for ontology development. It can be seen that the imported data which describe *2009*-*041D* are used directly as values of *data properties*, such as the values of the properties *size*, *rcs* and *brightness* etc. The relations of *2009*-*041D* and its related entities are described by object properties, such as the description “inOrbit orbitOf-1097454467”. Contextual data about external information describing *2009*-*041D* are represented through data properties describing relative objects, e.g., orbital data which describe *2009*-*041D* are used as values of data properties of *orbitOf*-*1097454467*. All sources of the data which describe *2009*-*041D* are annotated. In the lower right corner of Fig. 3, it can be seen that the source of the brightness datum of *2009*-*041D* is annotated as “data source: calsky.com”.

### Demo on Protégé of R-OBC

As pointed out in “Ontology-based classification” section, there are extra awards of R-OBC due to its conveniences in recording source of the knowledge and finding justifications for classifications (abbr. *classification justification*). A justification is a minimal set of knowledge deriving the conclusion for a classification. *Classification justification* can be done by the service *explanation* in Protégé. From Fig. 3, it can be seen that sources of the data which describe the RSO are recorded with annotation. Sources of the background knowledge and machine learning rules are also recorded in this way, so all the machine learning rules in these sets can be tracked.

**Fig. 4:**
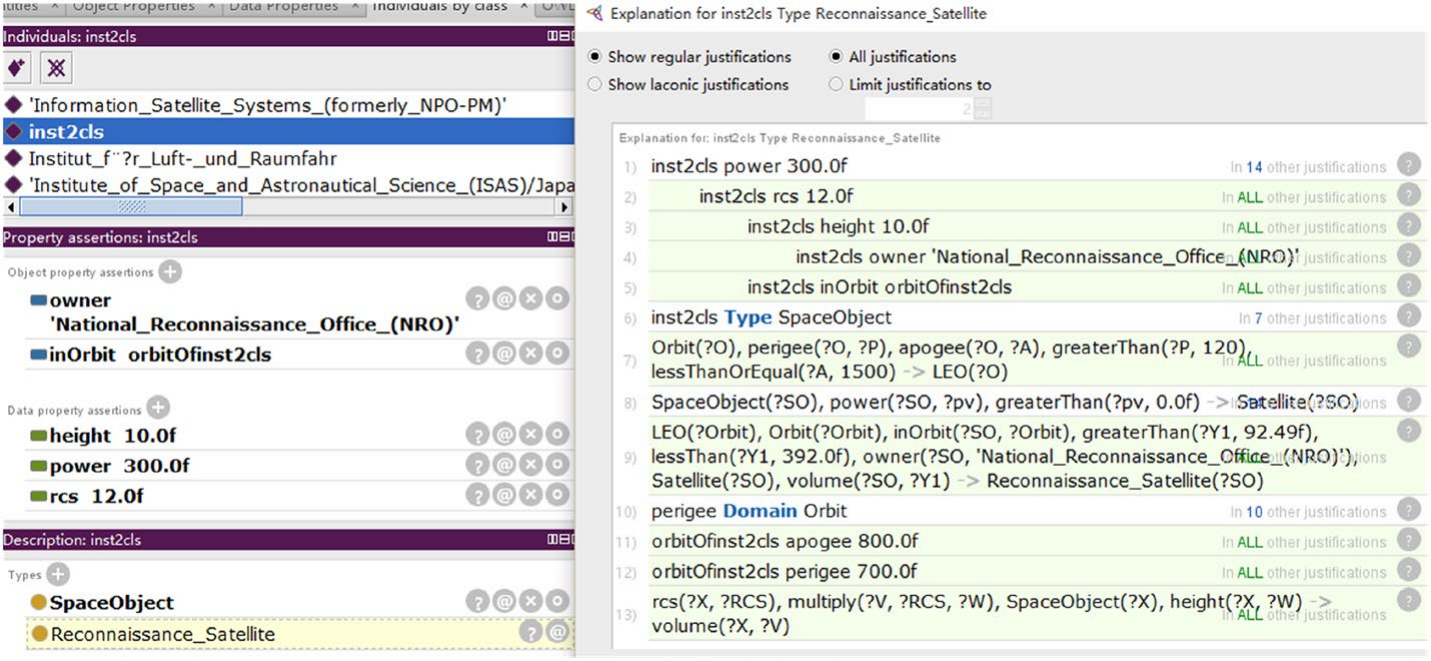
Finding justifications for a classification

Given an unlabelled RSO with data description, it can be classified via R-OBC and find a *classification justification*, by the following steps. Firstly, the RSO is represented in OntoStar as described in “Demo on Protégé of R-OBC” section. Then the ontology reasoning service *realization*, provided by the ontology reasoner pellet, is employed to compute the most specific class for the RSO. Finally, the service of *explanation* is employed to find a *classification justification*. An example of classifying an RSO named “inst2cls” (identified as *inst2cls* in the following) through R-OBC and finding a *classification justification* is shown in Fig. 4. The reasoning service *realization* is completed within 1840 ms.

**Fig. 5:**
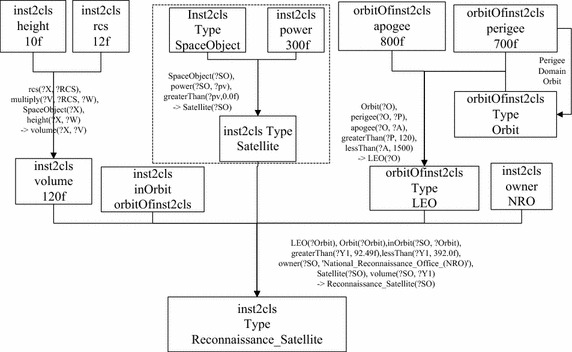
Justification parsed to tree-like structure

In Fig. 4, the imported data which describe *inst2cls* are shown in the middle box and lower box of the left part. The given knowledge about *inst2cls* contains information of *owner*, *orbit*, *rcs*, *height* and *power* of *inst2cls*, and the statement that *inst2cls* is an RSO. The statement that *inst2cls* is a *Reconnaissance_Satellite*, shown in the dot-line box of the lower left part, is derived from the given knowledge through ontology reasoning. A *classification justification* is found by the service *explanation*, and the *classification justification* is shown in the right part. With the *classification justification* being comprehensible, the deriving process from pre-condition to conclusion can be parsed into a intelligible tree-like structure, as shown in Fig. 5.

The tree-like structure in Fig. 5 records inference process for the classification, and helps users to understand how *inst2cls* is classified as *Reconnaissance_Satellite*. The traceable knowledge and intelligible justification in the transparent classification make every step of the classification by R-OBC able to be validated by experts, in order to make the classification assured. In Fig. 5 it can be seen that the tree-like structure inference process corresponds to RSO hierarchy in OntoStar. The inference process in the dot-line box shows *inst2cls* is classified as *Satellite* only with knowing *power* of *inst2cls*. This shows R-OBC is still able to classify an RSO with low specificity when other features/attributes are missing.

The main drawback of OBC is its classification time. As mentioned above, it comsumes 1840 ms to obtain the type of *inst2cls*. Even the loading time of the ontology can be ignored when the ontology is preloaded in memory, OBC still consumes 520 ms in average to classify an RSO using the general reasoner. A dedicated reasoner is required to enhance OBC’s computational efficiency for the classification when fast classification is a rigid demand.

### Weka-based test of R-OBC

The R-OBC system formed in “Demo on Protégé of R-OBC” section is integrated into WEKA, to take advantage of WEKA to compute indexes of testing results. R-OBC’s ability to perform space object classifications is measured by accuracy, FP rate, precision and recall.

Accuracy measures the actual performance of the system with regard to both correctly classifying and correctly rejecting objects. It is one of the most common metrics for a classifier. The higher accuracy a classifier get, the better the classifier. Accuracy is defined as following:

$${\text{accuracy}}\, = \,\frac{{{\text{true}}\;{\text{positives}}\,{ + }\,{\text{true}}\;{\text{negatives}}}}{{{\text{true}}\;{\text{positives}}\,{ + }\,{\text{false}}\;{\text{negatives}}\,{ + }\,{\text{true}}\;{\text{negatives}}\,{ + }\,{\text{false}}\;{\text{positives}}}}$$

False alarm rate is an important evaluation index in SSA (Laas-Bourez et al. 2011; Haith and Bowman 2014). The lower false alarm rate a classifier get, the better the method for object identification. False alarm rate is defined as the following (González and Dasgupta 2003; Stibor et al. 2005), which is the same as the FP rate calculated by WEKA:

$$false\;alarm\;rate = \frac{{{\text{false}}\;{\text{positives}}}}{{{\text{true}}\;{\text{negatives}} + {\text{false}}\;{\text{positives}}}}$$

Precision is the fraction of detected items that are correct. Recall is the fraction of items that were correctly classified among all the items that should have been classified. Other metrics such as F-Measure and Precision Recall Curve are determined by precision and recall.

“Ten-fold Cross Validations” (TCV) are performed on RSODS without the attributes *cospar_id* and *nord*_*id* by R-OBC and 7 classical machine learning classifiers, including C4.5 (Quinlan 2014), Bayesian Network (Friedman et al. 1997), Ripper (Cohen 1995), SVM (Keerthi et al. 2006), Random Forests (Breiman 2001), Backpropagation Neural Network (Erb 1995) and Logistic Model Trees (Landwehr et al. 2005). The baseline methods are implemented in WEKA as the following (a method in brackets correspond to one of its implementation in WEKA before the brackets respectively): J48 [C4.5], BayesNet [Bayesian Network], Jrip [Ripper], SMO [SVM], RandomForest [Random Forests], MultilayerPerceptron [Backpropagation Neural Network], and SimpleLogistic [Logistic Model Trees].

In the experiments, 50 trees are setup for RandomForest and 4 hidden layers are setup for MultilayerPerceptron (they will cost more than 250 s in learning for more trees or layers and more trees or layers enhance a little, so the number of trees and layers are not set too large). The resting baseline methods choose the default parameters in WEKA. In TCV, data are split into training data (90 %) and test data (10 %). To mimic the situation that some features of RSOs are missing because of the limited and uncertain observations, we remove an attribute in the test data. The results are shown in Table 3.

**Table 3:** Results of Ten-cross-fold validation on RSODS by WEKA

EI	M&D												
	R-OBC					J48				BayesNet			
	O[1]	O[2]	R	S	A	O	R	S	A	O	R	S	A
Accuracy	0.872	0.85	0.893	0.892	0.884	0.854	0.837	*0.707*	0.848	0.899	0.862	0.862	0.878
(W)FP rate	0.03	0.041	0.033	0.032	0.031	*0.124*	*0.157*	*0.437*	*0.119*	0.027	*0.14*	*0.137*	*0.085*
(W)precision	0.891	0.87	0.892	0.892	0.891	*0.809*	*0.79*	*0.609*	0.807	0.904	0.862	0.864	0.876
(W)recall	0.891	0.87	0.894	0.893	0.892	0.854	0.837	*0.707*	0.848	0.899	0.862	0.862	0.878
T (s)	0.87	4.1	0.36	0.29	0.46	3.74	3.58	3.64	3.95	0.27	0.11	0.11	0.13

EI	M&D											
	JRip				SMO				RandomForest (50-trees)			
	O	R	S	A	O	R	S	A	O	R	S	A
Accuracy	0.92	*0.864*	*0.817*	*0.838*	0.903	0.888	*0.395*	0.903	0.9	0.889	0.881	0.893
(W)FP rate	0.035	*0.084*	*0.228*	*0.191*	0.04	0.044	*0.251*	0.041	0.082	0.103	*0.136*	0.082
(W)precision	0.918	*0.847*	*0.816*	*0.843*	0.902	0.886	*0.527*	0.902	0.897	0.888	0.879	0.892
(W)recall	0.92	*0.864*	*0.817*	*0.838*	0.903	0.888	*0.395*	0.903	0.9	0.889	0.881	0.893
T (s)	3.7	3.23	3.31	3.64	3.67	3.47	3.65	3.70	*313.52*	*271.91*	*277.67*	*297.64*

EI	M&D							
	MultilayerPerceptron (4-hidden-layer)				SimpleLogistic			
	O	R	S	A	O	R	S	A
Accuracy	0.847	*0.46*	*0.155*	*0.740*	0.9	0.897	*0.746*	*0.742*
(W)FP rate	0.044	0.084	*0.085*	*0.223*	0.039	0.048	*0.267*	*0.373*
(W)precision	0.813	*0.691*	*0.667*	*0.668*	0.899	0.901	*0.683*	*0.635*
(W)recall	0.847	*0.46*	*0.155*	*0.740*	0.9	0.897	*0.746*	*0.742*
T (s)	*294.83*	*342.32*	*308.75*	*315.10*	*3532.5*	*3440.7*	*3479*	*3643.57*

Italic values indicate moderate negative significance

Underlined values indicate significant negative impact

Normal values indicate trivial significance

M&D: methods and data; (W)EI: (weighted average) evaluation index; O: original test data; R: unknown rcs in test data; S: unknown size in test data; A: unknown amr in test data; T: learning time for building model

In Table 3, indexes with prefix “(W)” are weighted average indexes computed by WEKA based on indexes of all classes. The rows O in Table 3 show results of evaluating the classifiers using original test data. R, S and A in Table 3 mean that the classifiers are evaluated by the test data missing rcs, size and amr respectively. R-OBC O[1] and R-OBC O[2] show *hierarchy guided learning* (corresponding to Algorithm 1) and *simple learning* (corresponding to Algorithm 1 without steps 3–11 and 20–23) of OBC evaluating by original test data respectively.

- Hierarchy guided learning in improving accuracy and efficiency

As can be seen in Table 3, *hierarchy guided learning* outperforms *simple learning* in all aspects. The OBC system gaining rules by J48 (R-OBC O[1] or R-OBC O[2]) outperforms J48 (J48 O) in most aspects. The above phenomena can be explained, that background knowledge plays roles in the process of learning and classification. *Hierarchy guided learning* makes the learned rules more accurate, and *background rules* take effect when learned rules fail to classify an RSO. Hierarchy guided learning (R-OBC O[1]) gains smaller decision trees in the above experiment (because only 406.8 rules in average are extracted from the trees in one validation, and 1061 rules in average are extracted from the tree of J48 in one validation). Smaller decision trees learned also are reflected by the less learning time of R-OBC than that of J48 (0.87 vs. 3.74 s).

(b) Robustness

Experiments are also conducted to test the robustness of R-OBC when one of the important attributes *rcs*, *size* and *amr* is missing in the test data. Comparisons in the following are all between evaluations using the data with one attribute missing and evaluations using the original test data. When the attribute *rcs* is missing in the test data, SMO, RandomForest and SimpleLogistic keep their performances in most aspects, with respect to original test data. Performances of J48, BayesNet and JRip drop a little in most aspects (in the range of 1.5–6 %) except a big increase (>10 %) in FP rate for BayesNet. The performances of MultilayerPerceptron reduce drastically. When the attribute *size* is missing in the test data, all classifiers’ performances reduce significantly, except for OBC and RandomForest. When the attribute *amr* is missing in the test data, performances of BayesNet, JRip, MultilayerPerceptron and SimpleLogistic reduce significantly, while other classifiers keep their performances.

R-OBC is robust when one of the attributes *rcs*, *size* and *amr* is missing. This can be explained that background knowledge plays roles. Firstly, *deduction knowledge* deduces the missing attributes from the known attributes precisely. Secondly, *background rules* take effect when learned rules fail to classify an RSO. The comparisons between R-OBC and classical classifiers on robustness are most obvious when the attribute *size* is missing. In fact, it is more difficult to obtain *size* than to obtain *rcs* of an RSO for us. It is common that there are some missing features when classify an RSO, so the classifiers have to be robust enough to tolerate such imperfect data.

(c) Enhanced flexibility

In terms of the learning part of building classifiers, as shown in Table 3, R-OBC and BayesNet take the least time (hundreds of milliseconds), JRip, J48 and SMO take the second least time (several seconds), while RandomForest, SimpleLogistic and MultilayerPerceptron take the most time (hundreds of seconds). It’s obvious R-OBC and BayesNet will be able to training classifiers faster than other machine learning methods, when the training data change. Therefore, once OntoStar has been built (although it demands much mannual work and is time-consuming), R-OBC is competitive in terms of learning the model. Especially, R-OBC benefits its unordered rule when part of the training data change, for adding or removing a classification rule will not influence other rules. For example, when new RSO data of a specific label are added to the training data, it is only necessary to learn classification rules of the label. For the datasets Norad_Catalog and UCS_Satellite, when new satellite data are added to UCS_Satellite, only classification rules of satellite need to be updated with the learning of UCS_Satellite.

(d) Other analyses

From Table 3 it can be seen that performances of R-OBC in most aspects are even better when the attribute *rcs*/*size/amr* is missing. When the attributes *rcs*, *size* and *amr* of an RSO are not missing, the deduced values (*rcs*, *size* and *amr*) will differ from that in test data, if the test data are biased or the *deduction knowledge* is not precise. At the same time, both the deduced value and the value in test data are used as the true value in the classification. Therefore, inconsistency occurs more frequently in classifications when there is no missing value. Because an RSO will not be classified by R-OBC when inconsistency occurs, R-OBC is able to classify more RSO when one of *rcs*, *size* and *amr* is missing.

## Conclusions

This paper presents a methodological framework for improving performances of classifying RSOs by harnessing ontology and machine learning techniques. An RSO is classified by reasoning of OntoStar and the classification process can be recorded. OntoStar is a hybrid knowledge base which contains both the latest domain knowledge in RSO classification advances and unordered machine learning rules. The learning processes are guided by the hierarchy of OntoStar when learning rules for OntoStar, in order to learn more accurate and comprehensible rules. The traceable knowledge is another extra award of OBC, which is explored through coding knowledge into ontology with annotations. The recorded classification process and traceable knowledge are comprehensible, making it possible to check every step of the classification, thus making the classification able to be validated by experts. All these efforts lead to the facilitation of SSA by accurate, robust and comprehensible RSO classification.

Three experiments are performed on collected RSO data. Multi-source RSO data are represented by OntoStar with sources of data recorded. Demo shows how an unclassified RSO is classified based on OntoStar, with the classification process specifically recorded. OBC and seven classical machine learning classifiers are employed for TCV on WEKA. Experiments show that, (1) OBC owns traceable knowledge and transparent classification processes, thus augments comprehensibility of classification; and (2) OBC is robust when some features are missing, and its learning part is fast. The main drawback of OBC is that, OBC consumes more time in classifying RSO than other machine learning classifiers. OBC consumes hundreds of milliseconds to classify an RSO in average, while other machine learning classifiers consumes less than a millisecond. Considering its contribution in dealing with the imperfect data, the disadvantage is still tolerant.

In future work, the authors will improve the efficiency of the reasoning procedure by developing a dedicated reasoner for OntoStar, making the most of the characteristics of OntoStar. In addition, ontology evaluation for ontology development, and introduction of multi-source unordered machine learning rules, are also expected to enhance the classification accuracy. As a further step, the authors will apply ontology evaluation and multi-source unordered machine learning rules to R-OBC. It will also be taken into consideration that background knowledge is learned for a fine-grain OntoStar. Despite the limitations mentioned above, the presented methodology can be extended and applied to the classification of various objects in other areas where robustness is required, as long as the training time and classifying time are not critical.

 Data and results of the experiments can be accessed from the following link: https://drive.google.com/folderview?id=0B6t3WjaQZyjWNXhnWFBUd0xjdmM&usp=sharing.

## Abbreviations

OWL: Ontology Web Language
SWRL: Semantic Web Rule Language
OBC: ontology-based classification
RSO: resident space objects
amr: area-to-mass ratio
rcs: radar cross section
NORAD: North American Air Defense Command
SSA: space situational awareness
UCS: Union of Concerned Scientists

## Footnotes

1 http://www.cs.waikato.ac.nz/ml/weka/.

2 https://github.com/Complexible/pellet.

3 http://www.ucsusa.org/nuclear_weapons_and_global_security/solutions/space-weapons/ucs-satellite-database.html.

4 http://satellitedebris.net/Database.

5 http://www.calsky.com/cs.cgi/Satellites.

6 https://www.space-track.org/.

7 http://www.standford.edu/protege.
